# Structural and Functional Impacts of Microbiota on *Pyropia yezoensis* and Surrounding Seawater in Cultivation Farms along Coastal Areas of the Yellow Sea

**DOI:** 10.3390/microorganisms9061291

**Published:** 2021-06-12

**Authors:** Arsalan Ahmed, Anam Khurshid, Xianghai Tang, Junhao Wang, Tehsin Ullah Khan, Yunxiang Mao

**Affiliations:** 1 Key Laboratory of Marine Genetics and Breeding (Ministry of Education), College of Marine Life Sciences, Ocean University of China, Qingdao 266003, China; arsalanahmed@163.com (A.A.); aanam7@gmail.com (A.K.); txianghai@ouc.edu.cn (X.T.); wjh@stu.ouc.edu.cn (J.W.); tehsinullahkhan0@gmail.com (T.U.K.); 2 Key Laboratory of Utilization and Conservation of Tropical Marine Bioresource (Ministry of Education), College of Fisheries and Life Science, Hainan Tropical Ocean University, Sanya 572022, China

**Keywords:** *Pyropia yezoensis*, microbiome, bacterial communities, Illumina NovaSeq, microbial composition, ribosomal RNA

## Abstract

*Pyropia yezoensis* is the most important commercial edible red algae in China, carrying a variety of resident microbes at its surface. To understand microbiome diversity, community structure, interactions and functions with hosts in this regard, thalli and seawater sampleswere collected from Yantai and Rizhao cultivation farms in the Yellow Sea. The thalli and seawater samples (*n* = 12) were collected and studied using an Illumina NovaSeq 6000 platform and 16S ribosomal RNA (rRNA) gene sequencing, along with the consideration of environmental factors. Bacterial communities in association with *P. yezoensis* and surrounding seawater were predominated by Cyanobacteria, Proteobacteria, and Bacteroidetes. The variability of bacterial communities related to *P. yezoensis* and seawater were predominantly shaped by nitrate (NO_3_), ammonium (NH_4_), and temperature. Cluster analysis revealed a close relationship between thalli (RTH and YTH) and seawater (RSW and YSW) in terms of the residing bacterial communities, respectively. PICRUSt analysis revealed the presence of genes associated with amino acid transportation and metabolism, which explained the bacterial dependence on algal-provided nutrients. This study reveals that the diversity of microbiota for *P. yezoensis* is greatly influenced by abiotic factors and algal organic exudates which trigger chemical signaling and transportation responses from the bacterial community, which in turn activates genes to metabolize subsequent substrates.

## 1. Introduction

*P. yezoensis* (laver), an economically important red seaweed, is a popular food and condiment that has a long history of cultivation and consumption in Asia [1] and is widely used around the world nowadays [2]. Conventionally, laver seaweeds have been considered as staple foods in limited regions of Asia, but an increased understanding of health benefits and the global availability of processed food products has led to a dramatic increase in consumption worldwide [3]. The growth of global seaweed aquaculture as a source of pharmaceuticals and biomaterials (e.g., by Alga Technologies and Cyanotech, etc.) is expected to contribute to the expansion of the laver industry [2,4]. Worldwide laver production has increased from 517,739 t/USD 945.1 billion in 1987 to 2,563,048 t/USD 2319.7 billion in 2017 [5]. China, South Korea, and Japan were the three largest producers, accounting for almost 99.87% of total world production in 2017 [5]. This represents the typical laver consumption in these countries, as well as the favorable regional aquaculture environmental conditions [6].

Marine microorganisms have been impacted by environmental impacts on several spatial scales, including changes in species distribution, habitat degradation, higher incidences of disease, species extinctions, and additionally alarming effects on the abundance, distribution, and function of marine planktonic microorganisms [7,8,9,10]. Macroalgae normally attract and promote beneficial bacterial species to colonize and multiply on their surface and, in turn, these epiphytic bacteria contribute to the algal life cycle by assisting the algae to counter the intrusion and colonization of harmful microorganisms by secreting secondary metabolites and/or antimicrobials [11]. Since the mid-twentieth century, the strong mutual interactions between bacteria and seaweeds have been studied, and several investigations have shown that green and brown macroalgae axenic cultures grow slowly or display anomalous morphogenesis [12,13,14,15,16,17,18]. Fries firstly documented this dependence for red algae while working with *Polysiphonia urceolata* [19], furthermore, in recent times, similar findings have been found in red algal species [20,21,22]. Investigations on the microbial makeup and diversity of edible red and brown algae, such as *Pyropia haitanensis*, *Porphyra umbilicalis*, and *Saccharina japonica*, have been undertaken prior to our findings [20,23,24]. Moreover, consumable red alga *Kappaphycus alvarezii* strains (brown, green) have been examined for the presence or absence of pathogenic bacterial strains [25]; however microbial community investigations of *P. yezoensis* are limited to a comparison between the healthy and diseased states (red rot), revealing a change in the inhabiting microbial community structure and abundance during infection [21,26]. These studies into the functional and compositional profiles of the microbial communities on and around macroalgae demonstrate the need of developing a comprehensive understanding of the microbiome of the macroalga *P. yezoensis*, which is both ecologically and economically vital.

The application of high-throughput DNA sequencing techniques to examine a microbial community’s genomic composition in a culture-independent mode has grown tremendously over the last few decades. This technique of high-resolution molecular sequencing, known as metagenomics, can be further sub-divided into two separate methodologies, namely 16S rRNA gene and shotgun metagenomic sequencing. To study bacterial phylogeny and taxonomy, the 16S rRNA gene sequencing method relies on 16S ribosomal RNA (rRNA) gene sequencing as a genetic marker, which consists of conserved hypervariable regions that can be used for bacterial identification [27]. Several research studies have focused on the microbial diversity of *Pyropia* using marker genes such as 16S rRNA genes, 18S rRNA genes, and internal transcribed spacer (ITS) genes [28,29]. A recent study investigated the change in bacterial population associated with *Pyropia* using 16S rRNA gene sequencing and found that there were over 300 operating taxonomic units (OTUs) distributed in approximately 15 microbial phyla [21].

Based on the previous studies, the present study aims to explore the microbial communities associated with *P. yezoensis* and the variations in surrounding seawater in different geographical locations. Moreover, this work aims to explore the contribution of abiotic parameters in driving variation in the structures of microbiota.

## 2. Materials and Methods

### 2.1. Study Sites and Sample Collection

**Figure 1:**
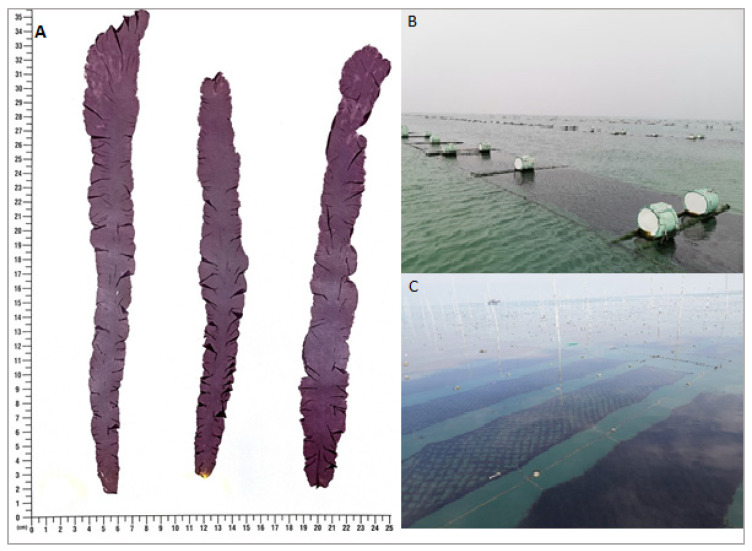
(**A**) *P. yezoensis* thalli. (**B**) Yantai cultivation farm. (**C**) Rizhao cultivation farm.

The *P. yezoensis* thalli and seawater samples were collected from two laver farms located in the coastal areas of Yantai (36°38′ N, 121°24′ E) and Rizhao (35°15′ N, 119°26′ E) of Shandong Province, China (Figure 1).

For the analysis of microbial communities, thalli samples (*n* = 3) and seawater samples (*n* = 3), were collected in triplicate from each farm, with a total of six thalli (*n* = 6) samples and six seawater (*n* = 6) samples from both regions, respectively. To make each of these biological replicates, neighboring triplicate samples (roughly 2 m away) were also collected and pooled (hundreds of pieces in total for thalli and 3 L in total for seawater). All the samples were brought to a laboratory within 4–6 h and were kept at 4 °C by soaking the thalli in collected seawater. Thalli samples were washed three times with sterilized seawater to remove loosely associated microbes and stored at −80 °C until further processing. Moreover, for each seawater replicate, approximately 600 mL was pre-filtered through a nylon mesh (200-μm pore size) to remove debris and subsequently filtered through a 0.22-μm mixed cellulose membrane (MerckMillipore, Darmstadt, Germany) to collect the microbial biomass.

For measuring environmental variables, seawater samples were collected in triplicate from both the Yantai and Rizhao farming sites. These samples were also used to evaluate the environmental stress on bacterial assemblages. The salinity, temperature, dissolved oxygen (DO), and pH values of the seawater were recorded during the sampling process with appropriate sensors at a depth of 50 cm, whereas the concentrations of phosphate, reactive silicate (RS), nitrate, nitrite, and ammonium were calculated using standard methods [30]. Three technical replicates were used for the determination of each variable for each sample.

### 2.2. DNA Extraction

For the extraction of bacterial community DNA from the thalli and seawater samples, the PrecellysTM-24 (Bertin Corp., Rockville, MD, USA) and DNeasy PowerSoil^®^ kit (Qiagen, Hilden, Germany) were used. Samples were shaken twice at the rate of 6800 shakes min^−1^ for 30 s with an interval of 5 min prior to DNA extraction [21]. Then, DNA was stored at −20 °C until amplification. DNA concentration and purity were monitored on 1% agarose gel and all DNA samples were diluted with sterile water to adjust the concentration to 1 ng/µL.

### 2.3. 16.S rRNA Gene Amplification

For the thalli and seawater samples, the V4 hypervariable region of 16S rRNA gene was amplified using the primer pairs of 515F (5′-GTGCCAGCM GCCGCGGTAAT-3′) and 806R (5′-GGACTACHVGGGTWTCTAA-3′) with the barcode. All PCR reactions were performed with 15 µL of the Phusion^®^ High-Fidelity PCR Master Mix (New England Biolabs), 0.2 µM of forward and reverse primers, and roughly 10 ng of template DNA. Thermal cycling for initial denaturation was performed at 98 °C for 1 min, followed by 30 cycles of denaturation at 98 °C for 10 s, annealing at 50 °C for 30 s, and elongation at 72 °C for 30 s, then finally 72 °C for 5 min. Each DNA sample was amplified in triplicate. The same 1× loading buffer (contained SYB green) volume was mixed with PCR products and electrophoresis on 2% agarose gel was used for detection. The PCR products were mixed in equal density ratios, and then the PCR products were purified with a Qiagen gel extraction kit (Qiagen, Hilden, Germany) in correct lengths.

### 2.4. Library Preparation and Sequencing

Using the TruSeq^®^ DNA PCR-Free sample preparation kit (Illumina, Inc., San Diego, CA, USA), sequencing libraries were generated following the recommendations of the manufacturer and index codes were added. On the Qubit@ 2.0 fluorometer (Thermo Scientific, CA, USA) and Agilent Bioanalyzer 2100 device, the library quality was evaluated. Finally, the library was sequenced on an Illumina NovaSeq 6000 platform.

### 2.5. Data Processing Based on Bioinformatics and Statistical Analysis

Based on their specific barcode, paired-end reads were assigned to the samples and trimmed by cutting off the barcode and primer sequence. Using the software FLASH v1.2.7 [31], paired end reads were merged and the splicing sequences were called raw tags. In order to obtain clean and high-quality tags, quality filtering of the raw tags was performed under specific filtering conditions [32] according to the QIIME v1.9.1 [33] quality control process. The tags were compared with a reference database (SILVA database) to detect chimera sequences using the UCHIME algorithm [34], and then the chimera sequences were removed [35]. Finally, the effective tags were obtained.

Uparse v7.0.1001 was used to perform the sequence analysis [36]. Sequences with ≥97% similarity were assigned to the same OTUs. For further annotation, representative sequences for each OTU was screened. The SILVA database [37] was used to annotate the taxonomic information for each representative sequence based on Mothur algorithm. In order to study the phylogenetic relationships of different OTUs and differences between the dominant species in different samples (groups), the MUSCLE software v3.8.31 was used to conduct multiple sequence alignment [38]. Finally, the data of each sample were normalized and the smallest amount of data in a sample was used as the standard.

Alpha diversity was applied to analyze the complexity of species diversity through calculating the observed species, Chao1, Shannon, Simpson, ACE, whole-tree PD, and Good’s coverage values with QIIME v1.7.0 and was displayed with R software v2.15.3.

For multi-sample comparative analysis (Beta diversity), QIIME v1.9.1 was used to calculate the UniFrac distance-based species complexity to evaluate differences between samples. Furthermore, R software v2.15.3 was used to draw the PCA and NMDS diagrams. For PCA analysis, the ade4 and ggplot2 packages were used. NMDS analysis was performed using the vegan package. To test the significance of the variations between the compositions of bacterial populations, Bray–Curtis distance-dependent dissimilarity tests (e.g., MRPP, ANOSIM, and Adonis) were used. Clustering with the unweighted pair-group method with arithmetic means (UPGMA) was performed as a type of hierarchical clustering to interpret the distance matrix using average linkage via the QIIME software (Version 1.9.1). For LEfSe analysis, the LEfSe software was used, and the LDA score was set to four as the default.

The PICRUSt (phylogenetic investigation of communities by reconstruction of unobserved states) software package was used to understand the potential genetic capabilities of the seaweed bacterial communities [39]. Redundancy analysis (RDA) was applied to reveal the relationships between bacterial communities and environmental variables in R using the vegan package.

Spearman correlation was performed between the abundance of bacterial community composition (top 35 genera) and the environmental factors in R by the correlation test function of the psych package and the pheatmap function in the pheatmap package [40], respectively.

## 3. Results

### 3.1. Physico-Chemical Properties of Seawater

The physicochemical properties of seawater samples are illustrated in Table 1. The NO_3_ and NH_4_ concentrations were significantly different (*p* < 0.05) between the regions of Yantai and Rizhao. A significant difference (*p* = 0.005) in temperature was also recorded, with higher values in Yantai as compared to Rizhao. Most of the other environmental variables were relatively stable and no significant (*p* < 0.05) differences were recorded (Table 1).

### 3.2. Bacterial Community Diversities

A total of 792,529 effective sequences of DNA were identified after the quality control process and 62,850–69,872 sequences per sample (mean ± standard deviation = 66,044 ± 1071) were obtained. According to the SILVA database, a total of 1202 OTUs were obtained and 92.43% of reads were classified at the phylum level and could be assigned into 19 and 20 phyla for the *P. yezoensis* and seawater samples, respectively. A petal diagram revealed that the bacterial OTUs associated with *P. yezoensis* of the Yantai and Rizhao regions had 55 core OTUs (Figure S1A), while 156 core OTUs were identified in the seawater samples (Figure S1B).

#### 3.2.1. Alpha Diversity

**Figure 2:**
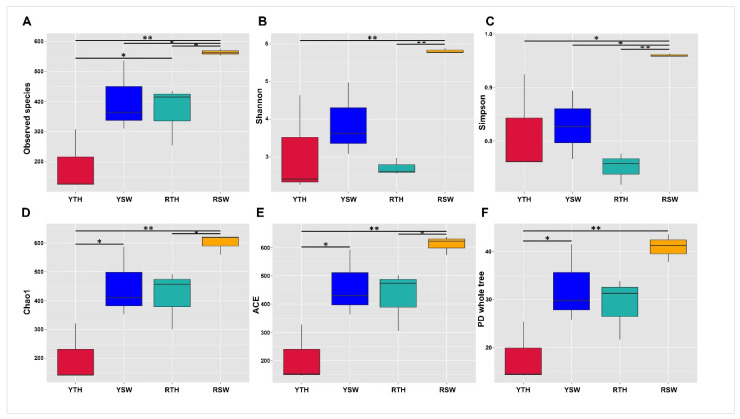
Comparisons of alpha diversity indices based on an unpaired Wilcoxon rank-sum test: (**A**) observed species; (**B**) Shannon; (**C**) Simpson; (**D**) Chao1; (**E**) ACE; (**F**) PD whole-tree. YTH, Yantai region thalli of *P. yezoensis*; YSW, Yantai region seawater sample group; RTH, Rizhao region thalli of *P. yezoensis*; RSW, Rizhao region seawater sample group. (* *p*-value < 0.05; ** *p*-value < 0.01).

Good’s coverage for all samples was found (greater than 99%), indicating that sequencing captured the majority of bacterial diversity in the samples (Table S1). Meanwhile, 62,452 randomly selected sequences (the lowest number in samples was 62,850) were used to evaluate the alpha diversity (i.e., ACE, Observed species, Shannon, Simpson, Chao1, and PD whole-tree) of each dataset (Figure 2 and Figure S2), whereas the alpha diversity indices showed that seawater features far higher bacterial diversity than *P. yezoensis*. According to the Wilcoxon rank-sum test community richness (Chao1, ACE), community diversity (Shannon, Simpson), and PD whole-tree results, significant differences (*p* < 0.05) with stochastic patterns were observed (Table 2); however, in observed species indices, the significant differences (*p* < 0.05) among all the sample groups were recorded (Table 2).

#### 3.2.2. Beta Diversity

**Figure 3:**
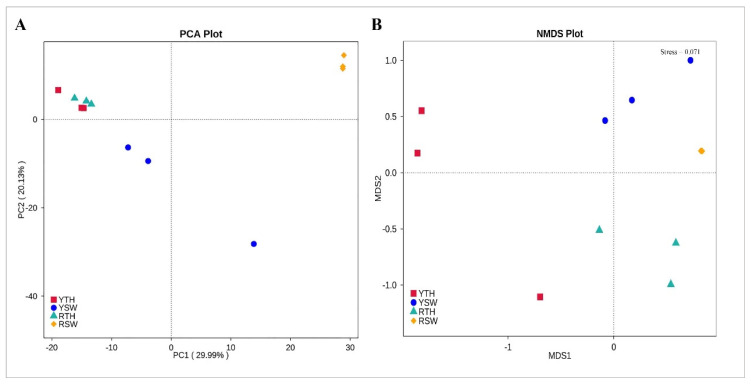
Relationships between individual datasets illustrated by (**A**) principal component analysis (PCA) and (**B**) nonmetric multi-dimensional scaling (NMDS).

**Figure 4:**
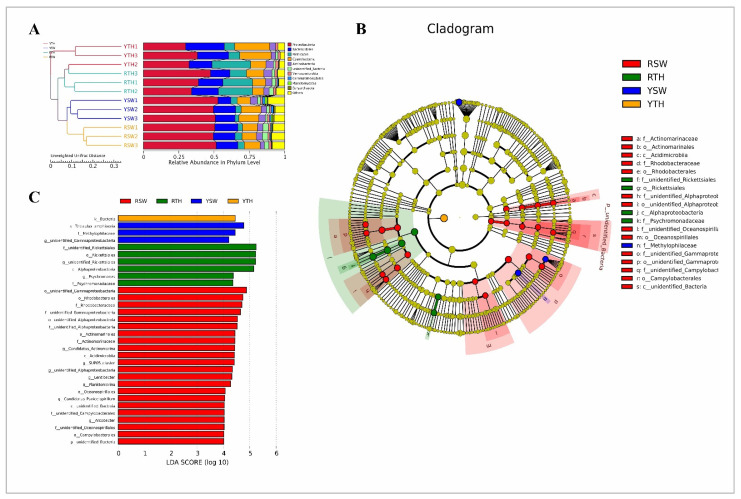
(**A**) UPGMA cluster tree analysis based on the unweighted UniFrac distance. The digital number symbolizes three biological replicates for each sample. (**B**) Evolutionary branch diagram of differential bacterial communities or species. (**C**) The LDA value (influence value of linear discriminant analysis) distribution histogram shows bacterial communities or species (Biomarker) with a LDA score greater than four.

For beta diversity interpretation, PCA-based Euclidean distances were used for the dimensionality reduction of multi-dimensional data, and NMDS ordination based on Bray–Curtis distances was performed to explain the clustering relationships between bacterial community datasets. In the PCA plot, *P. yezoensis* samples of Rizhao and Yantai regions clustered together, whereas seawater samples were distantly related with each other in both sampling regions (Figure 3A), whereas, in the NMDS plot, it should be noted that *P. yezoensis* samples of both the regions were clustered distantly from each other while, seawater samples clustered closely to each other (Figure 3B). The results of cluster analysis UPGMA based on UniFrac distance were consistent with the PCA. Thalli samples of both Rizhao and Yantai regions were clustered together, just like the seawater samples of both regions (Figure 4A).

Bray–Curtis-based Adonis, Anosim, and MRPP analysis values of *P. yezoensis* thalli and seawater samples were found to be greater than zero, thus indicating non-significant (*p* > 0.05) differences between groups rather than differences within groups (Table S2).

#### 3.2.3. Linear Discriminant Analysis Effect Size (LEfSe)

LEfSe (LDA effect size) is an analysis tool used to discover and interpret highly dimension biomarkers (genes, pathways, and taxa). Information on the differences in phylum, class, order, family, and genus levels among all bacteria is shown in the pie chart in Figure 4B. The LEfSe analysis used the species abundance data between sample groups to detect the species difference through the method of a rank-sum test and achieved dimensionality reduction through LDA (linear discriminant analysis) to evaluate the impact of the species differences. Overall, 32 biomarkers were identified with a LDA score >4. The maximum effect size in Rizhao *P. yezoensis* (RTH) was observed for family and order Rickettsiales of the phylum Proteobacteria. In contrast, the microbiota of Rizhao seawater (RSW) was characterized by a preponderance of microorganisms from the phylum Proteobacteria including orders of Gammaproteobacteria and Rhodobacterales, whereas in the Yantai region *P. yezoensis* (YTH), microbiota were characterized by bacterial biomarkers and the seawater microbiota (YSW) were dominated by the species *Teleaulax amphioxia* and family *Methylophilaceae*, belonging to the phyla of Cyanobacteria and Proteobacteria, respectively (Figure 4C).

### 3.3. Composition of Bacterial Community

**Figure 5:**
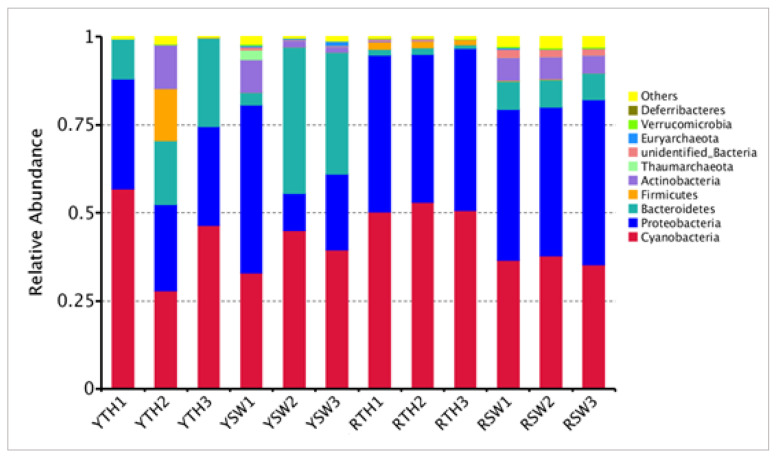
Bacterial community structures associated with *P. yezoensis* and seawater at the phylum and class levels. YTH, Yantai region *P. yezoensis* thalli; YSW, Yantai region seawater; RTH, Rizhao region *P. yezoensis* thalli; RSW, Rizhao region seawater.

The three taxa of bacterial communities that dominated in *P. yezoensis* and seawater were Cyanobacteria (42.7%), Proteobacteria (35.7%), and Bacteroidetes (13.6%), respectively (Figure 5). The percentages of dominant phyla associated with *P. yezoensis* of both regions were the following: Cyanobacteria, 47.5 ± 6.251%; Proteobacteria, 36.05 ± 13.407%; and Bacteroidetes, 9.9 ± 13.818% (Figure 5). The dominant OTUs of bacterial communities from both regions (water and thalli) are summarized in Table S3. For example, the datasets from Yantai *P. yezoensis* samples were dominated by OTU2 of unidentified Cyanobacteria (22%), followed by OTU1 of unidentified Rickettsiales (21%), and OTU811 of unidentified Cyanobacteria (15%), respectively. In the Rizhao *P. yezoensis* samples, OTU1 of unidentified Rickettsiales (34%) was the primary contributor, followed by OTU2 of unidentified Cyanobacteria (29%) and OTU811 of family unidentified Cyanobacteria (20%) (Table S3). Furthermore, SIMPER analysis revealed 12.4% dissimilarity index in these OTUs to differentiate the bacterial communities between two sampling regions (Table S4A).

In terms of the relative abundance and taxonomic level, the bacterial communities of seawater showed different profiles as compared to the *P. yezoensis* related communities of both regions. In seawater population, the dominant taxa were Cyanobacteria (37.8 ± 2.139%), Proteobacteria (35.25 ± 14.229%) and Bacteroidetes (17.25 ± 15.381%) (Figure 5). The prevalent OTUs contributed more than 10% in Yantai region seawater samples were OTU3 (*Tenacibaculum*, 24%), OTU2 (unidentified Cyanobacteria, 12%), and OTU5 (unidentified Cyanobacteria, 10%). In Rizhao seawater samples the dominated OTUs contributed more than 10% was only OTU4 (*Virgulinella fragilis*, 13%) while others showed less contribution (Table S3). SIMPER analysis revealed 16.7% dissimilarity index between these OTUs to differentiate each dataset (Table S4B).

The common bacterial taxa from Yantai and Rizhao *P. yezoensis* were represented by four OTUs with more than 1% contribution in their respective regions, including OTU1 (genus unidentified Rickettsiales), OTU2 (family unidentified Cyanobacteria), OTU4 (genus unidentified Cyanobacteria), and OTU811 (family unidentified Cyanobacteria). Common bacterial taxa from the seawater of more than 1% abundance from both the regions included nine OTUs: OTU1 (genus unidentified Rickettsiales), OTU4 (genus unidentified Cyanobacteria), OTU5 (genus unidentified Cyanobacteria), OUT6 (genus *Candidatus Actinobacteria*), OTU7 (*SUP05 cluster*), OTU8 (genus Alphaprotobacteria), OTU11 (order Gammaprotobacteria), OTU15 (family unidentified Gammaprotobacteria), and OTU310 (family *Methylophilaceae*) (Table S3).

### 3.4. PICRUSt Analysis on Metagenome Functions Prediction

Metagenome function prediction was carried out based on marker genes (such as 16S rRNA) by PICRUSt. At the primary level, eight categories of biological functional pathways, including metabolism, genetic information processing, environmental processing, organismal system, and human disease were summarized (Figure S3A). The most abundant predicted gene copy numbers were found for metabolism (48–51%), genetic information processing (16–20%), and environmental information processing (11–12%), respectively. It should be noted that genetic information processing and human disease were common in both thalli (RTH > YTH) groups, while cellular process and metabolism were prominent common functions in both seawater (RSW > YSW) groups (Figure S3A).

**Figure 6:**
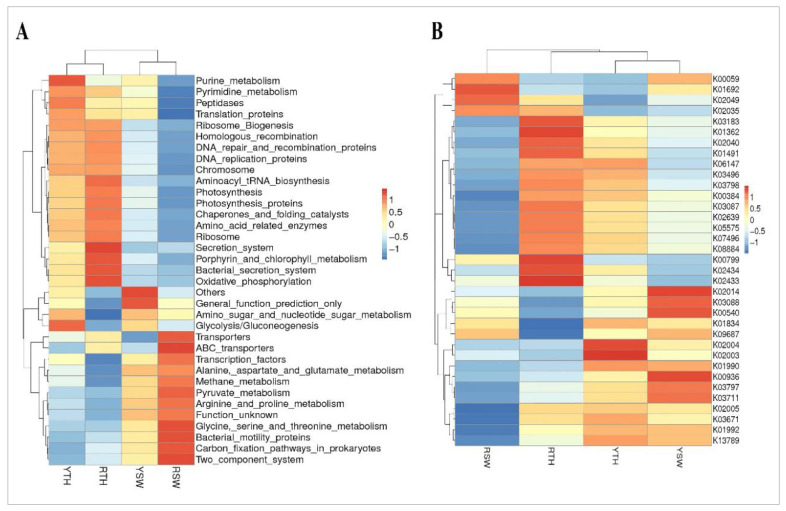
(**A**) Heat map display of the hierarchical clustering of the predicted KEGG ortholog (KEGG level 3) gene copy number (log10 transformed) of bacterial microbiota across all samples. (**B**) Cluster heat map display of KOs based on the predicted gene copy (log10 transformed) of bacterial microbiota across all sample groups. K00059 *fabG*, K01692 *paaF*, K02049 *ABC.SN.A*,K02035 *ABC.PE.S*, K03183 *ubiE*, K01362 *OVCH*, K02040 *pstS*, K01491 *folD*, K06147 *ABCB-BAC*, K03496 *parA*, K03798 *ftsH*, K00384 *trxB*, K03087 *rpoS*, K02639 *petF*, K05575 *ndhD*, K07496 *putative transposase*, K08884 *serine/threonine protein kinase*, K00799 *GST*, K02434 *gatB*, K02433 *gatA*, K02014 *TC.FEV.OM*, K03088 *rpoE*, K00540 *fqr*, K01834 *PGAM*, K09687 *Antibiotic transport system*, K02004 *ABC.CD.P*, K02003 *ABC.CD.A*, K01990 *ABC-2.A*, K00936 *pdtaS*, K03797 *ctpA*, K03711 *fur*, K02005 *ABC.CD.TX*, K03671 *trxA*, K01992 *ABC-2.P*, K13789 *GGPS*.

At the secondary level, multiple sub-functions were summarized, including biosynthesis of other secondary metabolites, signaling molecules and interaction, cell motility and amino acid metabolism (Figure S3B). The top 35 subfunctions are under discussion here, and cluster analysis showed that the thalli and seawater samples of both regions were closely related irrespective of the different sampling sites. Overall, 16 out of 35 sub-functions were common in seawater samples with the fluctuations in expression levels such as 5 of 16 sub functions showed decreased gene copy number in RSW as compared to YSW, rest followed revert patterns (Figure S3B). Most functions in thalli samples of both regions (YTH, RTH) were common except, metabolism and environmental adaptations with significant differences (*p* < 0.05) in the predicted gene copy number (Figure S4A). KO hierarchy level 3 indicated that thalli samples were closely related, and seawater samples were also clustered together. Most of the functions were common between seawater samples with variations in gene copy number following the trend RSW > YSW as shown in Figure 6A. In the thalli samples, there was a significant difference between plant-pathogen interaction (*p* ≤ 0.001), inositol phosphate metabolism (*p* = 0.01), and peptidases (*p* = 0.02). (Figure S4B).

Many studies have shown that the abundance and expression of functional genes in microbial communities can be precisely predicted by PICRUSt [41,42,43,44]. Furthermore, the abundant key genes in Yantai region seawater and thalli samples were K03711 *fur* (Fur family transcriptional regulator, ferric uptake regulator), K03797 *ctpA* (carboxyl-terminal processing protease), K00936 *pdtaS* (two-component system, sensor histidine kinase *PdtaS*), K02014 *FEV* (iron complex outer membrane receptor protein), K03088 *rpoE* (RNA polymerase sigma-70 factor), K00540 *fqr* (F420H(2)-dependent quinone reductase), K02004 *ABC* (putative ABC transport system permease protein), K02003 *ABC* (putative ABC transport system ATP-binding protein),and K01990 *ABC-2* (ABC-2 type transport system ATP-binding protein) (Figure 6B); however, it is worth mentioning that a significant difference was observed between YTH and YSW in the pathways of K03497 *parB* (*p* = 0.002), K00540 *ribBA* (*p* = 0.004), and K11717 *sufS* (*p* = 0.006) (Figure S5A).

The most prevalent predicted pathway genes in the Rizhao seawater (RSW) and thalli (RTH) samples were K0059 *fabG* (3-oxoacyl-[acyl-carrier protein] reductase), K01692 *paaF* (enoyl-CoA hydratase), K02049 *ABC.SN.A* (NitT/TauT family transport system ATP-binding protein), K03183 *ubiE* (demethylmenaquinone methyltransferase/2-methoxy-6-polyprenyl-1,4-benzoquinol methylase), K01362 *OVCH* (ovochymase), K00799 *GST*(glutathione S-transferase), K02434 *gatB* (aspartyl-tRNA(Asn)/glutamyl-tRNA(Gln) amidotransferase subunit B), and K02433 *gatA* (aspartyl-tRNA(Asn)/glutamyl-tRNA(Gln) amidotransferase subunit A). A significant (*p* < 0.05) distinction was found in many pathway genes of RTH and RSW (Figure S6). Meanwhile, thalli samples of Rizhao and Yantai (RTH, YTH) were clustered together, and the noticeable predicted common KO were K03183 *ubiE* (demethylmenaquinone methyltransferase/2-methoxy-6-polyprenyl-1,4-benzoquinol methylase), K01362 *OVCH* (ovochymase), K00799 *GST* (glutathione S-transferase), K02040 *pstS* (phosphate transport system substrate-binding protein), K01491 *folD* (methylenetetrahydrofolate dehydrogenase (NADP+), and K02005 *ABC.CD.TX* (HlyD family secretion protein); however, t-testing revealed a significant difference (*p* < 0.05) between the K00540 *fqr* (F420H (2)-dependent quinone reductase) and K013789 *GGPS* (eranylgeranyl diphosphate synthase, type II) pathways of thalli samples (Figure S5B). Overall, there were many common pathways activated in seawater and thalli samples of both regions, but their level of activation or gene copy number were randomly increasing or decreasing, respectively (Figure 6B).

### 3.5. Linkage between Bacterial Community Structures and Seawater Properties

**Figure 7:**
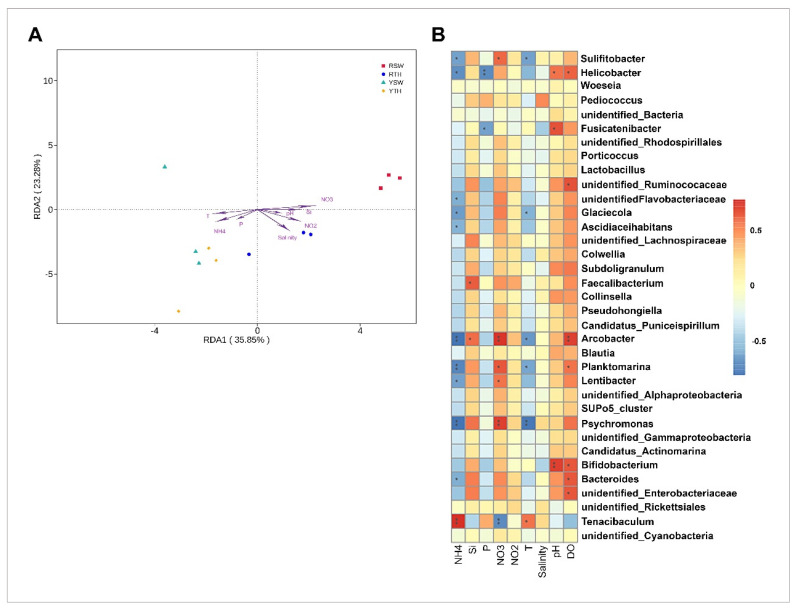
(**A**) Redundancy analysis (RDA) for bacterial community datasets associated with *P. yezoensis* and seawater. T, temperature; P, phosphate; RS, reactive silicate. (**B**) Spearman correlation analysis between seawater environmental factors and bacterial communities. The corresponding intermediate heat map value is the Spearman correlation coefficient r, and r > 0 is the positive correlation, r < 0 is negative correlation. * indicates significance where *p* < 0.05. ** indicates significance where *p* < 0.01.

The RDA analysis showed no significant effects (*p* > 0.05) for the nitrite, phosphate, pH, DO, salinity and reactive silicate concentrations on the bacterial community distributions in the Rizhao and Yantai regions. Ammonium (R^2^ = 0.646, *p* = 0.010), nitrate (R^2^ = 0.642, *p* = 0.006), and temperature (R^2^ = 0.411, *p* = 0.002) were significantly correlated with *P. yezoensis* and seawater-based bacterial communities (Figure 7A).

The Spearman correlation analysis showed stronger positive correlation of *Tenacibaculum* (OTU3) to ammonium concentrations, while *Phsychromonas* (OTU12), *Arcobacter* (OTU28), and *Planktomarina* (OTU13) were negatively correlated with ammonium concentrations, whereas *Arcobacter* showed a strongly positive correlation with nitrate and dissolved oxygen, which is the same as *Psychromonas* to nitrate. Other genera which showed strong correlations with seawater properties include *Bifidiobacterium* within Actinobacteria (OTU27), which was positively correlated with pH. On the other hand, *Helicobacter* within family *Helicobacteraceae* (OTU65) exhibited a strong negative correlation with phosphate; however, many other genera were also strongly correlated with seawater properties with positive and negative correlations (Figure 7B).

## 4. Discussion

Macroalgae perform essential roles in coastal ecosystems [45,46,47] are and known to contain diverse bacterial symbionts on their surfaces [48,49,50]. Microbial biofilm communities have showed effects on the health and normal functions of their hosts in multiple ways [51,52,53,54,55]. Increasing evidence suggests that such microbial community and host interactions and associations are specific [56,57,58]. These specific interactions represent profound implications for the prediction of microbial diversity and functional interactions in marine environments [57,59,60], which highlights the importance of investigating the interactions between hosts and microbial communities [61]. A variety of microbes resides on the surface of *P. yezoensis* [26] that differ in diversity, composition, and predicted functionality from each other and the surrounding water [62]. Significant scientific research has been aimed at studying the bacterial communities associated with seaweeds in recent decades to understand the interactions between bacteria and seaweed. [63]; however, the understanding of the diversity of microbial biofilm and complex relationships with their hosts is still in its beginnings. In this study, we provide insights into the variations in structure, composition, functional activities and algal-bacterial interactions of *P. yezoensis* and surrounding seawater associated microbial communities between two different sites, along with the roles of the physicochemical properties in driving the variability in the associated microbiota between the two regions.

## 4.1. Bacterial Community Structure and Abundance

The profile of the bacterial community on *P. yezoensis* and the surrounding seawater showed spatial resemblance and variation at the same time. Rizhao seawater showed richer and variable bacterial community structure as compared to Yantai seawater. The microbiota of *P. yezoensis* thalli showed an almost related bacterial population with slight variations. The alpha diversity indices reveal that seawater held much higher bacterial diversity than *P. yezoensis*, while the species richness and diversity indices showed significant (*p* < 0.05) differences among the seawater and thalli microbial communities of the same region (Figure 2). PCA and cluster analysis also confirmed the diversity of microbiota on *P. yezoensis* surface and seawater, respectively (Figure 3A and Figure 4A). These findings indicate that the bacterial communities may be shaped by the physicochemical properties of seawater. Moreover, the abundant phyla in the Rizhao and Yantai regions in *P. yezoensis* were Cyanobacteria, Proteobacteria, Bacteriodetes, and Firmicutes (Figure 5). As compared to other microbiota diversity studies, in our study, Cyanobacteria appeared as a dominant phylum, followed by Proteobacteria, which likewise dominated in the red seaweed *Laurencia dendroidea* [64]. These findings were consistent with Friedrich [65], who observed the bacterial species of the phyla Proteobacteria, Bacteroidetes, and Cyanobacteria in macroalgal species. Furthermore, the bacterial abundance belonging to Firmicutes and Bacteroidetes has been found in different red and green seaweeds, such as *Caulerpa racemosa, Bryopsis pennata Caulerpa cylindracea, Bryopsis hypnoides, Monostroma hariotii*, *and Ulva intestinalis* [66,67,68]. Whole-genome sequencing of *P. haitanensis* microbiota revealed the highest abundance of Proteobacteria (54.64%) and Bacteroidetes (37.92%) [23].

Members of Alphaproteobacteria and Gammaproteobacteria have a worldwide spread in coastal waters [69,70], while other commonly occurring marine taxa include Bacteroidetes, Actinobacteria, Plankomycetes, and Chloroflexi [61,70,71]. In our research, structural alterations were exhibited by bacterial population within seawater, which were particularly revealed by the discrepancies in abundance of prevalent OTUs distributed within the dominant phylum/class. (Table S3). For example, the prevalent OTUs in Yantai and Rizhao seawater samples were OTU3 *Tenacibaculum*. As the genus *Tenacibaculum* has a cosmopolitan distribution within saltwater, local distributions of *Tenacibaculum* spp. are largely unknown [72]. Moreover, non-pathogenic *Tenacibaculum* spp. has been described for red algae, tunicates, tidal sediments, seawater, mollusks, and crustaceans [72,73,74,75]. While members of the genus *Tenacibaculum* perform many functions in marine environments, including the decomposition of biopolymers like cellulose derivatives, xylan, agar, and chitin, some have algae-dissolving activities and also play a crucial role in carbon metabolism [22,76,77]. OTU4 (*Virgulinella fragilis*) and OUT6 (*Candidatus Actinomarina*), were also dominant in the seawater samples from both regions. Bacterial communities of *Candidatus Actinomarina* have already been reported in seawater previously [78] and are known to play a key role in organic matter processing (i.e., transport and degradation) in oceans [79]. Rizhao seawater also had a higher abundance for the OTU7 *SUP05* cluster. Members of the *SUP05* clade reported in marine waters and are supposed to contribute directly by the successive reduction of nitrate (NO_3_^−^) to nitrogenous gases (N_2_O or N_2_) [80]. Various studies have also identified the occurrence of these genera in algae and surrounding seawater [22,50,81]. Our study results are in agreement with Wei et al., who identified similar profile of bacterial communities in *P. yezoensis* and surrounding seawater by 16S rRNA gene sequencing approach [82]. Nevertheless, it should be acknowledged that different algal phyla show varying proportions of these bacterial taxa. This may be due to the limitations of the datasets; however, the results pose interesting considerations for the definition of “core” groups of bacterial communities. Additionally, functionally distinct algae (e.g., from different phyla) might not be expected to have a similar core.

## 4.2. Effects of Abiotic Factors on Community Structure

Apart from biotic variables like holobiont, the presence of grazer species [83,84,85,86] and natural abiotic variables is also responsible for an increase or decrease in the growth and marine diversity of algal species [87]; however, an increasing body of evidence indicates that seaweed interactions with microbiota and epifauna, habitat, growth, development, and abundance depend on the variations of abiotic factors like salinity, pH, temperature, sun radiation, and nutrients [84,85,86,88,89,90,91]. In this study, ammonium (R^2^ = 0.646, *p* = 0.010), nitrate (R^2^ = 0.642, *p* = 0.006), and temperature (R^2^ = 0.411, *p* = 0.002) were strongly associated with *P. yezoensis* and seawater-borne bacterial assemblages (Figure 7A). Many studies have reported the role of temperature in determining microbial populations and their functional activities by effecting metabolic niches [92,93,94,95]. Similarly, Li et al. stated that regional environmental factors (e.g., pH and temperature) influence the patterns of microbial community in *Carassius auratus* culture ponds [96]. In this study, ammonium and nitrate showed a significant (*p* < 0.05) correlation with the identified microbial communities. Shilova et al. reported that the addition of NH_4_ and NO_3_ caused a strong shift in the abundance and compositions of microbial communities [97].

## 4.3. Functional Profiles Of Bacterial Communities

The evolving consensus is that the structures of bacterial communities on macroalgae are primarily determined by functional genes rather than taxonomic or phylogenetic compositions, but also in conjunction with the microenvironment provided by the physiological and biochemical properties of the algal host [61,83,98]; however, the role of functional genes associated with the bacterial community assembly on seaweed surfaces lacks adequate information. In an attempt to gain insight in this regard, we used Phylogenetic Investigation of Communities by Reconstruction of Unobserved States (PICRUSt) to analyze and predict the functional capabilities of bacterial communities inhabiting *P. yezoensis* in thalli and seawater samples. The proteins of seaweed contain huge amounts of essential amino acids such as arginine, alanine, glycine, proline, aspartic, and glutamic acids [99]. This describes the greater abundance of bacterial genes associated with amino acid metabolism in this study (Figure S3B). The presence of genes related with aspartate, alanine, and glutamate metabolism elucidated the bacterial reliance on algae producing amino acids as an adaptive mechanism of bacteria in *Pyropia*. Furthermore, multiple biosynthetic genes were also identified, including the genes for biosynthesis of amino acids and genes for the production of terpene. Approximately 50,000 terpenoid metabolites have been identified in fungi and terrestrial and marine plants; however, only a few of these broadly occurring metabolites have been isolated from prokaryotes [100]. The synthesis of terpene in *Dinoroseobacter* and *α-Proteobacteria Loktanella* has been reported by Schulz and Dickscha [101]. Furthermore, Wei et al. recently stated that the production of terpene in red alga is accomplished through MTPSLs (microbial terpene synthase-like genes), which are found to be phylogenetically distinct from typical plant terpene synthases and their emergence in Rhodophyta can presumably be defined by horizontal gene transfer from bacteria [82].

Quorum sensing (QS) is a common mechanism for communication and works through chemical signaling and has gained and exclusive consideration by marine ecologists [102]. It is understood that quorum sensing induces downstream changes in gene regulation and alters biological functions such as biofilm formation, sporulation, bacterial conjugation, and bioluminescence [103,104,105]. In this study, we have identified genes related to the peptide/nickel transport system (K02035) that are involved in the quorum sensing pathway (K02024). This might be an interesting factor to understand interactions in bacterial communities. Moreover, several studies have reported quorum sensing mechanisms in different marine bacteria [106,107,108]. Meanwhile, side by side to this, we have identified a large number of genes for signaling pathways such as K00936, K02035, and K02040 (Figure 6B). According to previous reports it is thought that component systems are the only recognized channels for environmental sensing in bacteria. Nevertheless, thanks to recent technological advances in genome sequencing, genetic approaches have discovered the presence of eukaryote-like serine/threonine protein kinases (STPKs) and phosphatases in different prokaryotes, which includes many pathogenic bacteria such as *Yersinia* spp. [109,110], *Listeria monocytogenes* [111,112], *Mycobacterium* [113,114], *Pseudomonas aeruginosa* [115], *Enterococcus faecalis* [116], and *Staphylococcus *aureus** [117,118,119]. The sensor/signaling proteins of the serine/threonine protein kinase (STPK) family of virulence determining pathogenic bacteria perform the dual role of intuiting the environment and destabilizing the specific host defense system. STPKs have the capability to sense a wide array of signals and coordinate multiple cellular processes in order to generate an appropriate response [120]. A small number of transport genes and vitamin B12 genes were also detected the presence indicate that associated microbiota produces regulatory compounds and transport them to algal host it is stated previously in many studies that algae acquire vitamin B12 through a symbiotic relationship with bacteria [121,122,123].

In our study, the association of complex bacterial communities with algae and seawater is evident. The presence of variable functional profile, community structure and composition can have positive and negative effects on their interaction with macroalgae. Complex interactions have been justified by Goecke et al. [11,124] and Florez et al. [125], whereas the induction of morphological development [126], antimicrobial activity [127,128], and negative effects, for example, of pathogenic bacteria [129], and the presence of bacteria responsible for the degradation of macroalgae, also include in these interactions [130].

## 5. Conclusions

In summary, our study provides evidence for complex and diverse associations of microbial communities with *P. yezoensis* and surrounding seawater, displaying an exceptional metabolic, transport, and biosynthetic functions that are essential for favorable interactions and adaptability to the ecological environment. Meanwhile, the microbiota may play a vital role in secondary metabolite production and nutrient cycling, which are substantially influenced by algae metabolites and the physicochemical characteristics of seawater, which triggers the initiation of chemical signaling and transport pathway responses with requisite genes to metabolize subsequent substrates. In return, bacteria produce regulatory compounds and secondary chemical metabolites for algal growth, morphogenesis, and defense. To improve the understanding of bacterial diversity and mutualistic interactions under natural environmental conditions, research using broader sample data based on deep metagenomic sequencing and meta transcriptomes of microbial communities is required. Future research should emphasize the chemical signaling environment, genetic exchange, and metabolic interactions between *P. yezoensis* and its harboring microbiota to understand the effects of the functional profile of the host and symbiont in shaping the microbial diversity of the niche.

## Figures and Tables

**Table 1:** Environmental variables of Yantai and Rizhao seawater samples.

Environmental Variables	YSW	RSW	*p*-Value
DO (ppm)	15.86 ± 0.161	16.39 ± 0.44	0.123
Temperature (°C)	6.8 ± 0.205	5.9 ± 0.200	0.005 *
Salinity (%)	3.28 ± 0.097	3.33 ± 0.118	0.684
pH	6.90 ± 0.200	7.09 ± 0.262	0.639
NH_4_ (μmol/L)	2.527 ± 0.260	0.85 ± 0.429	0.004 *
Si (μmol/L)	5.39 ± 0.263	6.30 ± 0.921	0.175
NO_3_ (μmol/L)	4.70 ± 0.806	11.301 ± 0.901	0.001 *
NO_2_ (μmol/L)	0.751 ± 0.032	0.836 ± 0.069	0.130
Phosphate (μmol/L)	0.177 ± 0.015	0.178 ± 0.009	0.911

* Data are represented as the mean ± standard deviation. Bold *p*-values indicate a significant difference between the two regions. Means compared using unpaired *t*-testing and significance is determined as *p* < 0.05.

**Table 2:** Alpha diversity indices differences among the sample groups.

Sample Groups	Whole-Tree PD	Chao1, ACE	Simpson	Shannon	Observed Species
RTH-YTH	0.091	0.071	0.244	0.071	0.047 *
RSW-YSW	0.155	0.071	0.047 *	0.071	0.024 *
RTH-RSW	0.053	0.029 *	0.002 *	0.004 *	0.012 *
YTH-YSW	0.031 *	0.029 *	0.492	0.854	0.024 *

* *p*-values less than 0.05 are indicated with bold characters.

## Data Availability

Data will be provided upon request.

## Supplementary Materials

The following are available online at https://www.mdpi.com/article/10.3390/microorganisms9061291/s1, Figure S1: Petal diagrams showing the total number of OTUs and the number of shared OTUs across all *P. yezoensis* (A) and seawater (B) datasets. Figure S2: Refraction curves of observed species, Shannon, chao1 and PD whole-tree. Figure S3: KEGG pathway annotation (A) KEGG level 1 functional annotations (B) KEGG level 2 functional annotations. Figure S4: *t*-test (A) KEGG secondary level YTH-RTH (B) KEGG level 3 YTH-RTH. Figure S5: T-test (A) YTH-YSW KO hierarchy level (B) YTH-RTH KO hierarchy level. Figure S6: *t*-test of RTH-RSW KO level. Table S1: Average values of alpha diversity indices. Table S2: Significance differences in *Pyropia yezoensis* and seawater associated bacterial community structures, based on Bray–Curtis dissimilarities. Table S3: Average relative abundance of top 30 OTUs in *Pyropia yezoensis* and seawater sample groups. Table S4: SIMPER analysis (A) Dissimilarity contribution of bacterial OTUs within *Pyropia yezoensis* datasets. (B) Dissimilarity contribution of bacterial OTUs within seawater datasets.
