# Supplementary material for: Structural and Functional Impacts of Microbiota on Pyropia yezoensis and Surrounding Seawater in Cultivation Farms along Coastal Areas of the Yellow Sea
Source: Microorganisms. 2021 Jun 12;9(6):1291. doi: 10.3390/microorganisms9061291 (PMC8231614; doi:10.3390/microorganisms9061291)
Supplement: Supplementary file 1 [file microorganisms-09-01291-s001.zip › Supplementary material/Figure SI-S6.pdf]

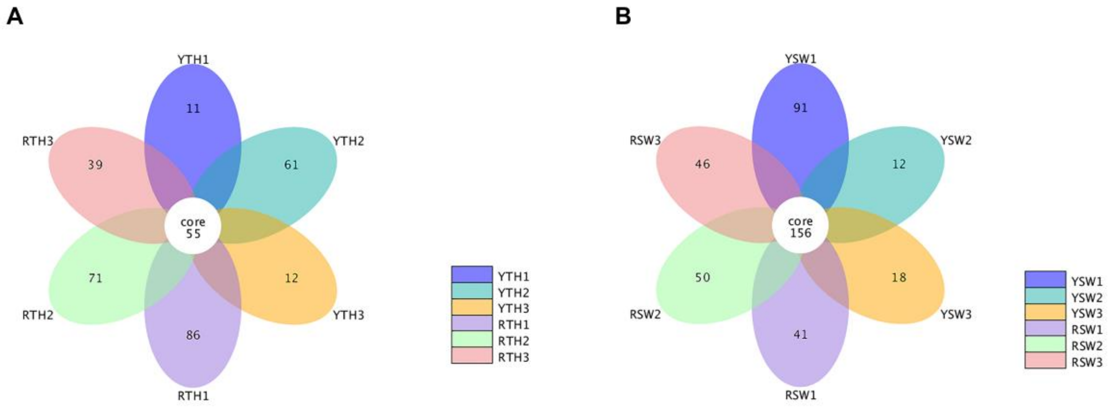


**Figure S1.** Petal diagrams showing the total number of OTUs and the number of shared OTUs across all *P. yezoensis* (A) and seawater (B) datasets.

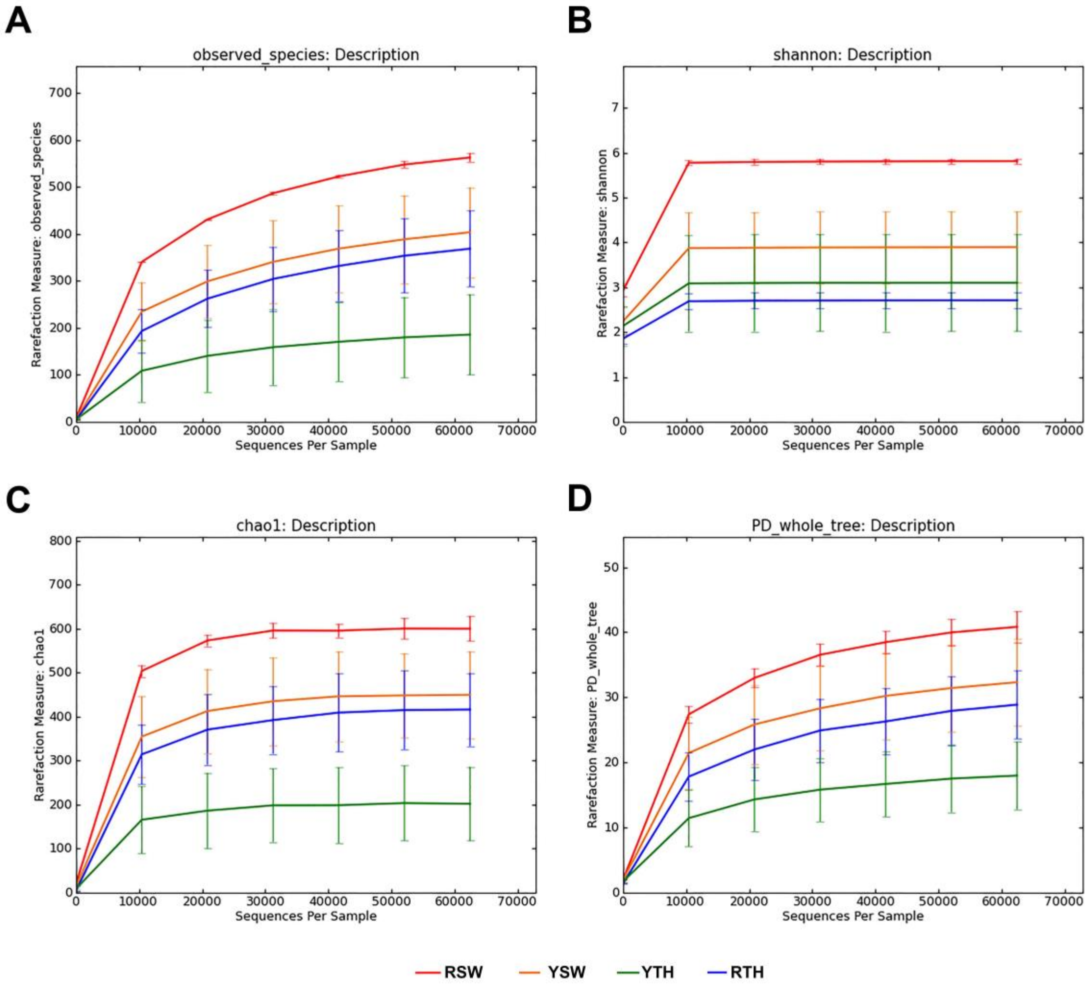


**Figure S2.** Refraction curves of observed species, Shannon, chao1 and PD whole-tree.

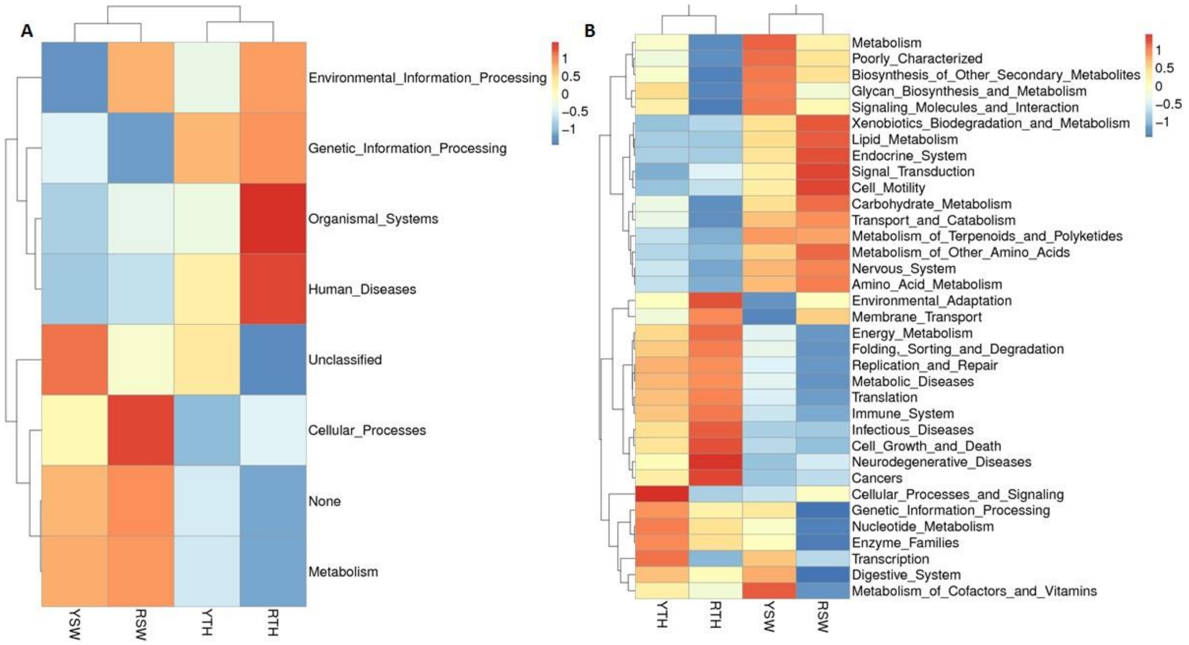


**Figure S3.** KEGG pathway annotation (A) KEGG level 1 functional annotations (B) KEGG level 2 functional annotations.

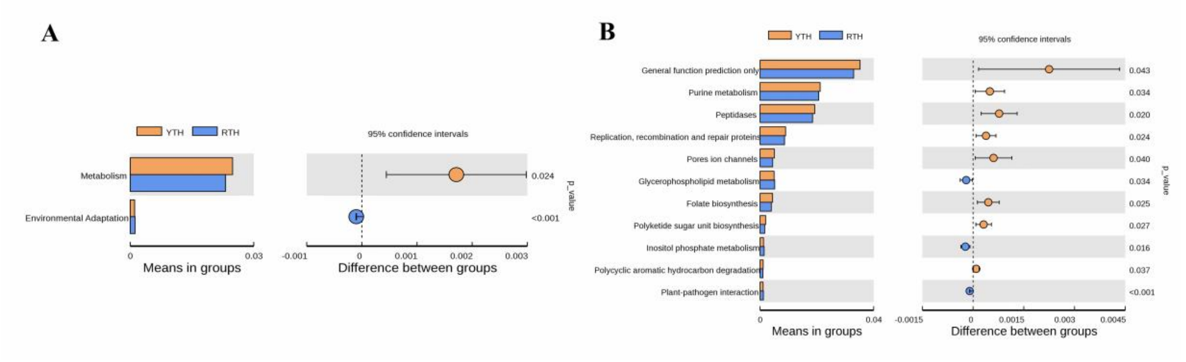


**Figure S4.** *t*-test (A) KEGG secondary level YTH-RTH (B) KEGG level 3 YTH-RTH.

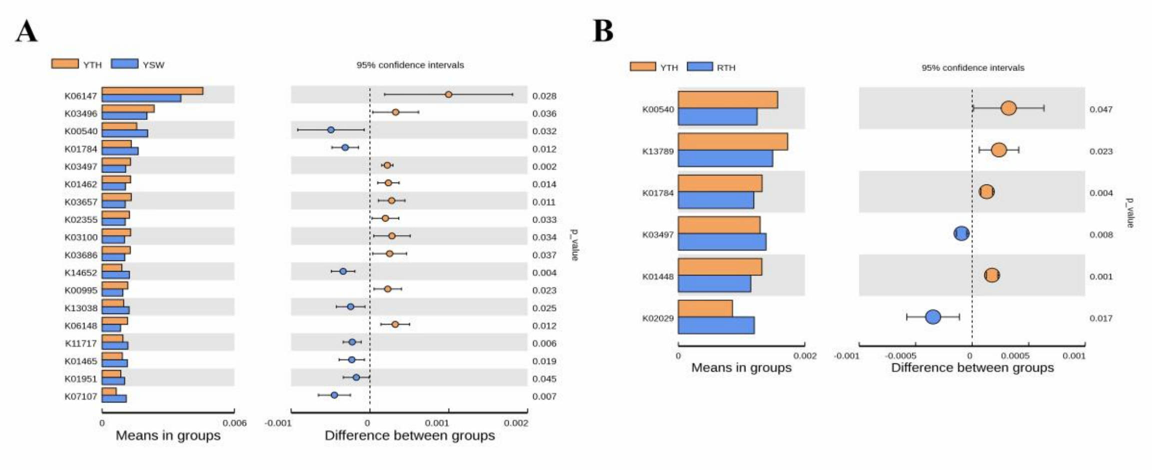


**Figure S5.** T-test (A) YTH-YSW KO hierarchy level (B) YTH-RTH KO hierarchy level.

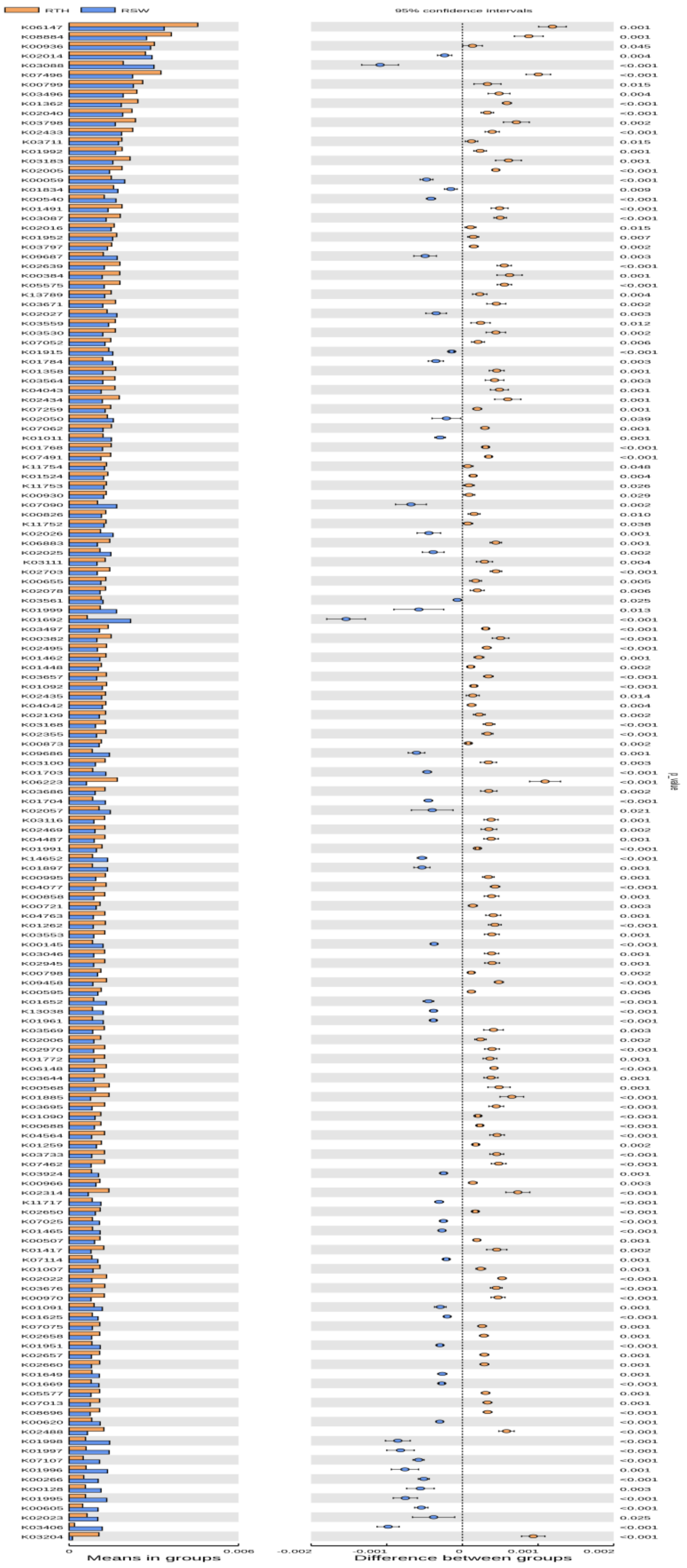

**Figure S6.** *t*-test of RTH-RSW KO level.
