# Supplementary material for: Structural and Functional Impacts of Microbiota on Pyropia yezoensis and Surrounding Seawater in Cultivation Farms along Coastal Areas of the Yellow Sea
Source: Microorganisms. 2021 Jun 12;9(6):1291. doi: 10.3390/microorganisms9061291 (PMC8231614; doi:10.3390/microorganisms9061291)
Supplement: Supplementary file 1 [file microorganisms-09-01291-s001.zip › Supplementary material/Table S1.pdf]

Table S1. Average values of Alpha diversity indices.

	Observed species	Shannon	Simpson	Chao1	ACE	Goods_coverage	PD_whole_tree
RSW	562	5.813	0.960	600.388	611.734	0.998	40.836
RTH	368	2.712	0.751	416.604	427.090	0.999	28.887
YSW	403	3.896	0.829	450.071	462.092	0.999	32.336
YTH	185	3.101	202.222	202.222	209.177	1.000	17.993
