# Supplementary material for: Structural and Functional Impacts of Microbiota on Pyropia yezoensis and Surrounding Seawater in Cultivation Farms along Coastal Areas of the Yellow Sea
Source: Microorganisms. 2021 Jun 12;9(6):1291. doi: 10.3390/microorganisms9061291 (PMC8231614; doi:10.3390/microorganisms9061291)
Supplement: Supplementary file 1 [file microorganisms-09-01291-s001.zip › Supplementary material/Table S2.pdf]

Table S2. Significance differences in *P. yezoensis* and seawater associated bacterial community structures, based on Bray-Curtis dissimilarities. Bold p values indicates the significant differences between the two sample groups.

**Sample**	**Anosim**		**MRPP**		**Adonis**	
**Groups**	R-value	P-value	A	P-value	R^2	P-value
**RTH-YTH**	0.5556	0.1	0.1338	0.1	0.69853	0.1
**RSW-YSW**	0.3333	0.2	0.3708	0.1	0.47825	**0.001389***
**RTH-RSW**	1	0.1	0.8101	0.1	0.02151	**0.001389***
**YTH-YSW**	0.1111	0.4	0.08217	0.1	0.71845	0.2
